# Ethyl Pyruvate Directly Attenuates Active Secretion of HMGB1 in Proximal Tubular Cells via Induction of Heme Oxygenase-1

**DOI:** 10.3390/jcm8050629

**Published:** 2019-05-08

**Authors:** Min Suk Seo, Hye Jung Kim, Hwajin Kim, Sang Won Park

**Affiliations:** 1 Department of Pharmacology, Institute of Health Sciences, Gyeongsang National University College of Medicine, Jinju 52727, Korea; smith26@daum.net (M.S.S.); hyejungkim@gnu.ac.kr (H.J.K.); hwajin1@gmail.com (H.K.); 2 Department of Internal Medicine, Samsung Changwon Hospital, Changwon 51353, Korea

**Keywords:** acute kidney injury, ethyl pyruvate, heme oxygenase-1, high mobility group box-1, ischemia and reperfusion, proximal tubular cells

## Abstract

Renal ischemia reperfusion (IR) is a main cause of acute kidney injury leading to high morbidity and mortality during postoperative periods. This study investigated whether ethyl pyruvate (EP) protects the kidney against renal IR injury. Male C57BL/6 mice were treated with vehicle or EP (40 mg/kg) 1 h before ischemia and the plasma creatinine (Cr) levels and tubular damage were evaluated after reperfusion. EP attenuated the IR-induced plasma Cr levels, renal inflammation and apoptotic cell death, but the effect of EP was abolished by pretreating Zinc protoporphyrin (ZnPP), a heme oxygenase (HO)-1 inhibitor. HO-1 is a stress-induced protein and protects the kidney against IR injury. EP increased significantly HO-1 expression in the proximal tubular cells in vivo and HK-2 cells in vitro. Inhibition of PI3K/Akt pathway and knockdown of Nrf2 blocked HO-1 induction by EP. High mobility group box 1 (HMGB1) secretion was assessed as an early mediator of IR injury; plasma HMGB1 were significantly elevated as early as 2 h to 24 h after reperfusion and these were attenuated by EP, but the effect of EP was abolished by ZnPP. EP also reduced HMGB1 secretion stimulated by TNF-α in HK-2 cells, and the inhibition of PI3K/Akt and knockdown of HO-1 blocked the effect of EP. Conclusively, EP inhibits the active secretion of HMGB1 from proximal tubular cells during IR injury by inducing HO-1 via activation of PI3K/Akt and Nrf2 pathway.

## 1. Introduction

Acute kidney injury (AKI) is characterized by a rapid decrease of kidney function, ranging from hours to weeks, resulting in diagnostic features of the increased blood urea nitrogen and plasma creatinine (Cr) levels. The incidence of AKI has been reported as 2–7% in hospitalized patients and an incidence of 5–10% in intensive care unit (ICU) population and the incidence rates are steadily increasing. [1,2]. Despite advances in the clinical management of disease, AKI is associated with an increased risk of death in hospitalized individuals, particularly in those admitted to the ICU with the mortality rate exceeding 50%. So, AKI, therefore, is associated with increased length of hospital stay and financial costs [2,3]. AKI can be induced by numerous causes which can be prerenal azotemia, intrinsic renal AKI, and postrenal AKI. Intrinsic renal AKI can be caused by tubular, glomerular, interstitial, and vascular damage. Acute tubular necrosis (ATN) is the most common cause of AKI and present in 50% of all hospital-acquired AKI [4].

Ischemia and reperfusion (IR) is a pathological condition characterized by an initial restriction of blood supply followed by a subsequent perfusion and reoxygenation [5,6]. In the tissues exposed to an ischemic condition, restoration of blood flow or reoxygenation is frequently associated with an exacerbation of tissue injury and a profound inflammatory response, which is called as a reperfusion injury [6]. The proximal tubules of the outer medulla are more vulnerable than the thick ascending limb of the medulla due to a high oxygen demand [7]. The IR injury induces ionic imbalance due to ATP and oxygen depletion in proximal tubular cells, leading to polarity loss and subsequent cell death, and ultimately renal dysfunction [1]. Thus, protection of proximal tubular cells during IR is therapeutically important and development of pharmacological agents that protect proximal tubules is under active investigation. 

High mobility group box 1 (HMGB1) is identified as a DNA-binding protein that functions as a cofactor for proper transcriptional regulation, whereas extracellular HMGB1 acts as a potent proinflammatory cytokine contributing to diverse inflammatory diseases [8]. HMGB1 is passively released from necrotic cells to the extracellular space or actively secreted upon external stimuli such as endotoxin (lipopolysaccharide, LPS) or tumor necrosis factor (TNF-α) from activated immune cells, such as macrophages [9]. Recent studies have reported that HMGB1 is released by renal parenchyma or inflammatory cells during renal IR and plays a critical role in signal transduction following injury [10]. HMGB1 expression in the kidney is increased after renal IR injury and administration of neutralizing antibody to HMGB1 reduces tubular apoptosis and inflammation [10,11], suggesting a therapeutic potential of HMGB1-targeting agents.

Heme oxygenase (HO) is a rate-limiting enzyme that degrades heme to biliverdin, carbon monoxide (CO) and ferrous iron [12]. Biliverdin is converted to bilirubin by bilirubin reductase; bilirubin has an antioxidant and anti-complement effect [13,14]. HO plays a key role in iron recycling and homeostasis. In addition, HO-1 derived CO is implicated in vascular biology; CO is a potent vasodilator and inhibitor of platelet aggregation that protects against cardiovascular diseases [15,16]. Thus, HO provides cellular protection through its antioxidant, anti-inflammatory, and anti-apoptotic properties. The HO family has two distinct isoforms, HO-1 and HO-2 sharing ~42% of amino-acid homology. HO-1 is stress-induced and typically expressed in the spleen, liver, and kidney, while HO-2 is constitutive expressed with high levels in the brain [12]. HO-1 is the most studied for its cytoprotective roles. HO-1 is induced by various stimuli, including cytokines/endotoxin, heat shock, heavy metals, and ischemic or oxidative stress. The therapeutic effect of HO-1 has been shown in many pathological conditions, including atherosclerosis, hypertension, endotoxic shock, and acute renal injury [17,18]. The renal HO-1 expression is relatively low in physiological conditions, but rapidly induced by acute renal insults such as ischemia or nephrotoxin, particularly in proximal tubules. HO-1 staining is prominent in tubular epithelial cells and infiltrating macrophages in many human renal diseases [19]. Thus, HO-1 is critical for the maintenance of renal function by protecting tubular epithelial cells under renal injury. HO-1 expression levels determine the course of renal disease and HO-1 deficiency worsens the pathophysiology. Plasma levels of HO-1 can be a biomarker for AKI and pharmacologic induction of HO-1 is used in preclinical models of renal disease [20]. 

Ethyl pyruvate (EP) is a simple derivative of pyruvate, and has antioxidant and anti-inflammatory properties; thus, EP has been studied in various preclinical disease models [21]. EP has been reported to attenuate lethal systemic inflammation in septic mice by inhibiting HMGB1 release [22]. EP has shown to inhibit proinflammatory response to LPS (endotoxin) in macrophages and improves survival in septic mice by inducing HO-1 [23]. However, not many studies have reported the effect of EP on renal IR injury, or the protective mechanism of EP. This study investigated the protective effect and mechanism of EP on mice subjected to renal IR and HK-2 cells treated with TNF-α.

## 2. Experimental Section

### 2.1. Animals

Male C57BL/6 mice (7-weeks old) were purchased from Koatech (Pyeongtaek, Korea) and maintained at the animal facility of Gyeongsang National University (GNU). All animal experiments were conducted in accordance with the National Institutes of Health guidelines for laboratory animal care with the approval of the Institutional Board of Animal Research at GNU. Mice were maintained with a 12-h light/dark cycle and provided freely with water and standard chow.

### 2.2. Surgical Procedure of Renal IR Injury

The mice were divided into six groups: (1) sham-operated mice (Veh sham, *n* = 4); (2) sham-operated mice treated with EP (Sigma, St. Louis, MO, USA; 40 mg/kg; intraperitoneal injection) (EP sham, *n* = 4); (3) mice subjected to IR injury (Veh IR, *n* = 8); (4) mice pretreated with EP 1 h prior to IR (EP IR, *n* = 8); (5) mice pretreated with Zinc protoporphyrin (ZnPP; Sigma; 10 mg/kg, intraperitoneal injection (i.p.)) 2 h prior to IR (ZnPP + Veh IR, *n* = 8), and (6) mice pretreated with ZnPP 1 h prior to EP and treated with EP 1 h prior to IR (ZnPP + EP IR, *n* = 8). The mice were anesthetized with zoletil (0.5 mg/kg; Virbac Laboratories, Carros, France) and placed supine on a heating pad under a heat lamp to maintain body temperature. After abdominal incision, a microvascular clamp was placed on the left renal pedicle for 25 min and a contralateral kidney was removed. During the ischemic period, mice were remained hydrated with warm saline. After removing the clamp, the incision was sutured. The sham mice were subjected to right nephrectomy without clamping. Mice were sacrificed and blood and kidney were collected. Plasma creatinine levels were measured by using Pure Auto S CRE-N (Daiichi Sankyo, Tokyo, Japan). The kidneys were rapidly frozen in liquid nitrogen or fixed in 10% formalin.

### 2.3. Cell Culture and Treatment

HK-2 human proximal tubular epithelial cells were maintained in a 1:1 mixture of Dulbecco’s modified Eagle medium (Thermo Fisher Scientific, Waltham, MA, USA)/Kaighn’s modification of Ham’s F-12 medium (F-12K; Thermo Fisher Scientific), supplemented with 10% fetal bovine serum and 1% penicillin/streptomycin (Hyclone Laboratories, Logan, UT, USA). HO-1 or Nrf2-specific siRNA and scrambled siRNA were purchased from Bioneer (Daejeon, Korea). The cells were transfected with Lipofectamine (Invitrogen, Carlsbad, CA, USA) reagents and incubated with siRNA (50 nM) for 24 h. Cells were pretreated with LY294002 (PI3K inhibitor, 10 µM), PD98059 (ERK inhibitor, 50 µM), SP600125 (JNK inhibitor, 40 µM), SB203580 (p38 inhibitor, 10 µM) or vehicle for 1 h, and treated with EP (25 mM) or vehicle as indicated in figure legends. Cells were treated with TNF-α (R&D Systems, Minneapolis, MN, USA) to mimic an IR-induced injury in vitro. 

### 2.4. Cell Viability 

Cell viability was assessed by 3-(4,5-dimethylthiazol-2-yl)-2,5-diphenyltetrazolium bromide (MTT) assay. The cells were incubated with MTT solution (final 0.1 mg/mL) and incubated at 37 °C for 4 h. Then, the supernatant was removed and formazan crystals were dissolved in dimethyl sulfoxide. Absorbance at 570 nm was measured using an Infinite 200 microplate reader (Tecan Austria GmbH, Grödig, Austria). Lactate Dehydrogenase (LDH) released into media was measured by using a LDH assay kit (Promega, Madison, WI, USA) according to the instruction. 

### 2.5. H&E Staining and TUNEL Assay

Kidney tissue was fixed in 10% formalin for 24 h, treated for paraffin embedding, and sectioned at 5 µm. Then, the sections were stained with H&E (Sigma) by a standard protocol. TUNEL assay was performed by an in situ cell death detection kit (Roche Molecular Biochemicals, Mannheim, Germany) according to the instruction. Images were captured using a CKX41 light microscope (Olympus, Tokyo, Japan). The number of apoptotic cells was counted from five microscopic fields (400×) per each section. 

### 2.6. Immunohistochemistry

The paraffin sections were deparaffinized and boiled in 10 mM of sodium citrate buffer for 40 min. Endogenous peroxidase activity was blocked with 0.3% hydrogen peroxide and nonspecific binding sites were blocked with 10% normal goat serum. The sections were incubated with primary antibodies (anti-Ly-6B.2 from Bio-Rad and anti-HMGB1 from Abcam) overnight at 4 °C, and incubated with biotinylated secondary antibody (Vector Laboratories, Burlingame, CA, USA) at room temperature for 1 h. Then, the sections were washed, incubated in an avidin-biotin-peroxidase complex solution (ABC solution; Vector Laboratories), and developed by using a 3,3’-diaminobenzidine (DAB) Peroxidase Substrate Kit (Vector Laboratories). The sections were counterstained with hematoxylin and immunostained images were obtained by a CKX41 light microscope (Olympus). 

### 2.7. Immunofluorescence Staining

Sections were blocked in 2.5% normal goat serum, and incubated with primary anti-HO-1 antibody (Santa Cruz Biotechnology) and fluorescein isothiocyanate (FITC)-conjugated secondary antibody (Invitrogen). HK-2 Cells were fixed with 4% paraformaldehyde for 20 min, and nonspecific binding was blocked in PBS-T including 1% bovine serum albumin at room temperature for 1 h. The cells were incubated with primary antibodies (anti-Nrf2 or anti-HMGB1 from Abcam) at 4 °C overnight, washed, and subsequently with FITC-conjugated secondary antibody (Invitrogen) for 1 h at room temperature. After mounting, immunostained images were visualized using a Fluoview 1000 (IX-81) confocal microscope (Olympus).

### 2.8. Western Blot Analysis

Kidney tissues or HK-2 cells were homogenized in ice-cold RIPA buffer with protease inhibitors (Thermo Fisher Scientific), sonicated, and incubated for 20 min on ice. After centrifugation, the supernatant was transferred to a clean tube and protein concentration was measured by a Bio-Rad protein assay kit (Bio-Rad, Hercules, CA, USA). Nuclear and cytoplasmic fractions were isolated using NE-PER Nuclear and Cytoplasmic Extraction Reagent kit (Thermo Fisher Scientific) according to the instructions. The proteins were separated using SDS-PAGE, transferred to PVDF membranes, and the membranes were incubated with primary antibodies. HO-1, p-Akt, and Akt antibodies were purchased from Cell Signaling Technology; anti-proliferating cell nuclear antigen (PCNA) antibody was purchased from Santa-Cruz Technology; HMGB1 and Nrf2 antibodies were purchased from Abcam. Then, then membranes were incubated with horseradish–peroxidase-conjugated secondary antibodies (Bio-Rad, Hercules, CA, USA) and with ECL substrates (Bio-Rad) for protein detection by the ChemiDoc XRS+ System (Bio-Rad). 

### 2.9. Reverse Transcription-Polymerase Chain Reaction (RT-PCR)

Total RNA was extracted using TRIzol Reagent (Thermo Fisher Scientific) and converted into cDNA using the AccuPower^®^ RT Premix (Bioneer) according to the instructions. One g of RNA was reverse transcribed. The mRNA levels were normalized to GAPDH. The primer sets are listed as follows: TNF-α, 5′-TACTGAACTTCGGGGTGATTGGTCC-3′ and 5′-CAGCCTTGTCCCTTGAAGAGAACC-3′; interleukin-6 (IL-6), 5′-GTTGTGCAATGGCA-ATTCTG-3′ and 5′-GCCACTCCTTCTG-TGACTCC-3′; monocyte chemoattractant protein-1 (MCP-1) 5′-ACCTGCTGCTACTCATTCAC-3′ and 5′-TTGAGGTGGTTGTGGAAAAG-3′; macrophage inflammatory protein-2 (MIP-2), 5′-TCCA-GAGCTTGAGTGTGACG-3′ and 5′-CTTTGGTTCTTCCGTTGAGG-3′; GAPDH 5′-GCTGAG-TATGTCGTGGAGTCTA-3′ and 5′-CATACTTGGCAGGTTTCTCCAG-3′. The PCR products were analyzed by electrophoresis on a 1.5% agarose gel and detected under UV light. 

### 2.10. Statistical Analysis

Statistical difference was assessed by two-tailed student t-test to compare two groups or by one-way analysis of variance (ANOVA), followed by Tukey post-hoc multiple comparison tests, to compare multiple groups. The values are expressed as the mean ± standard error of the mean (SEM). A *p*-value <0.05 was considered statistically significant.

## 3. Results

### 3.1. EP Protects the Renal Function and Structure against Renal IR Injury through HO-1

**Figure 1:**
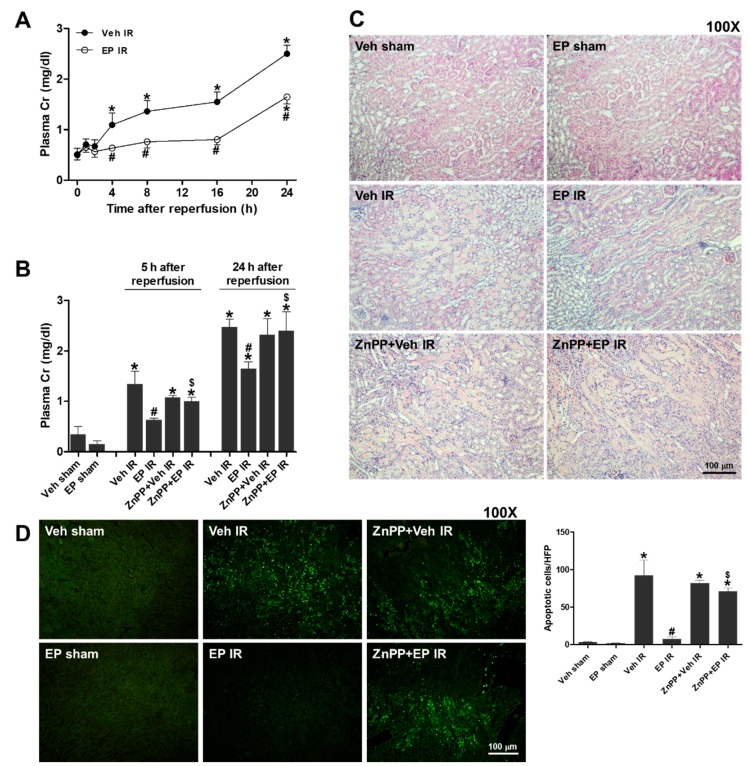
Ethyl pyruvate (EP) reduces plasma creatinine (Cr) levels and renal pathology during renal ischemia reperfusion (IR). C57BL/6 mice were subjected to either a sham-operation or 25 min of renal ischemia and subsequent reperfusion. EP (40 mg/kg) and ZnPP (10 mg/kg) were given intraperitoneally (i.p.) to mice at 1 h and 2 h prior to ischemia, respectively. (**A**) Plasma Cr levels at 0–24 h after reperfusion in the mice treated with vehicle or EP. (**B**) Plasma Cr levels at 5 or 24 h after renal IR. (**C**) Representative images of H&E staining of renal sections from the mice at 24 h after reperfusion. (**D**) Representative images of TUNEL staining of sections from the mice at 24 h after reperfusion, and apoptotic cells were counted. Data are presented as the mean ± standard error of the mean (SEM). * *p* < 0.05 vs. Veh sham, ^#^ *p* < 0.05 vs. Veh IR, ^$^ *p* < 0.05 vs. EP IR. Scale bar, 100 µm.

To determine the effect of EP on renal function impaired during IR injury, the mice were subjected to 25 min of renal ischemia and plasma were collected at 1, 2, 4, 8, 16 and 24 h after reperfusion. Plasma creatinine (Cr) levels of EP-treated mice were significantly reduced after 4 h of reperfusion compared to those of sham mice (Figure 1A). The mice pretreated with ZnPP, an inhibitor of HO-1, showed no protective effect of EP on plasma Cr levels at 5 and 24 h after reperfusion (Figure 1B). To examine histological changes, renal sections were stained with H&E (Figure 1C). Renal IR caused severe tubular necrosis, case formation, and tubular dilation, particularly at the cortex-medullary junctions. The results indicate that HO-1 mediated the effect of EP on protecting renal function and structure against renal IR injury.

To determine the effect of EP on apoptotic cell death during renal IR injury, the sections were subjected to the TUNEL assay (Figure 1D). IR induced a substantial increase in the number of apoptotic tubular cells at 24 h after reperfusion. EP significantly reduced the number of apoptotic cells, but the effect of EP was abolished by ZnPP pretreatment. The results indicate that EP attenuated tubular cell death during renal IR injury through HO-1.

### 3.2. EP Reduces Neutrophil Infiltration and Proinflammatory Cytokines during Renal IR Injury through HO-1

**Figure 2:**
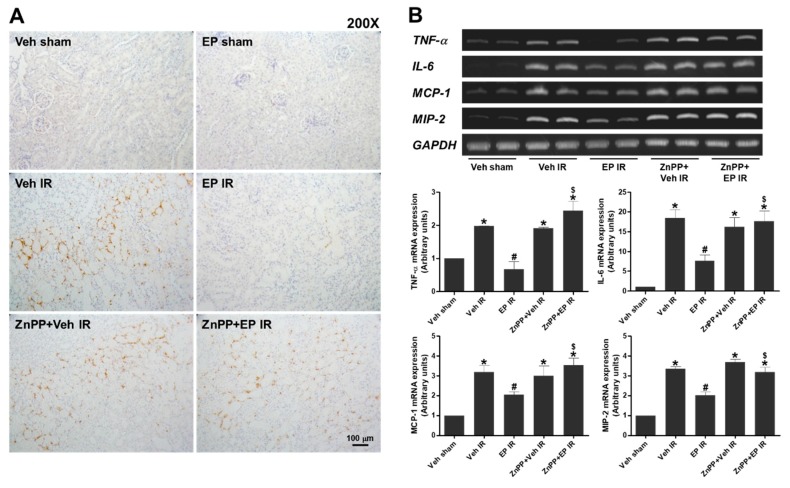
EP attenuates renal inflammation and apoptotic cell death during renal IR. Mice were subjected to either a sham-operation or 25 min of renal ischemia and 24 h of reperfusion. EP (40 mg/kg) and ZnPP (10 mg/kg) were given i.p. to mice at 1 h and 2 h prior to ischemia, respectively. (**A**) Neutrophil infiltration was assessed by polymorphonuclear leukocyte (anti-Ly-6B.2) staining of renal sections. (**B**) Representative RT-PCR bands of indicated genes from renal tissues and the semi-quantitative mRNA levels were shown. Data are presented as the mean ± SEM. * *p* < 0.05 vs. Veh sham, ^#^ *p* < 0.05 vs. Veh IR, ^$^ *p* < 0.05 vs. EP IR. Scale bar, 100 µm.

To determine the effect of EP on IR-induced renal inflammation, renal sections were subjected to immunohistochemical staining of polymorphonuclear leukocytes. Renal IR caused neutrophil infiltration particularly at cortex-medullary junctions, which was significantly reduced by EP. However, the effect of EP was blocked by ZnPP pretreatment (Figure 2A). The mRNA levels of proinflammatory cytokines and chemokines were measured in the kidney tissues at 5 h after reperfusion (Figure 2B). EP significantly reduced the levels of TNF-α, IL-6, MCP-1 and MIP-2 compared to vehicle. ZnPP pretreatment blocked the effects of EP on reducing these mRNA levels. The results indicate that EP reduced IR-induced renal inflammation through HO-1. 

### 3.3. Plasma HMGB1 Levels Were Rapidly Increased at an Early Reperfusion and the HMGB1 Secretion Was Attenuated by EP through HO-1

**Figure 3:**
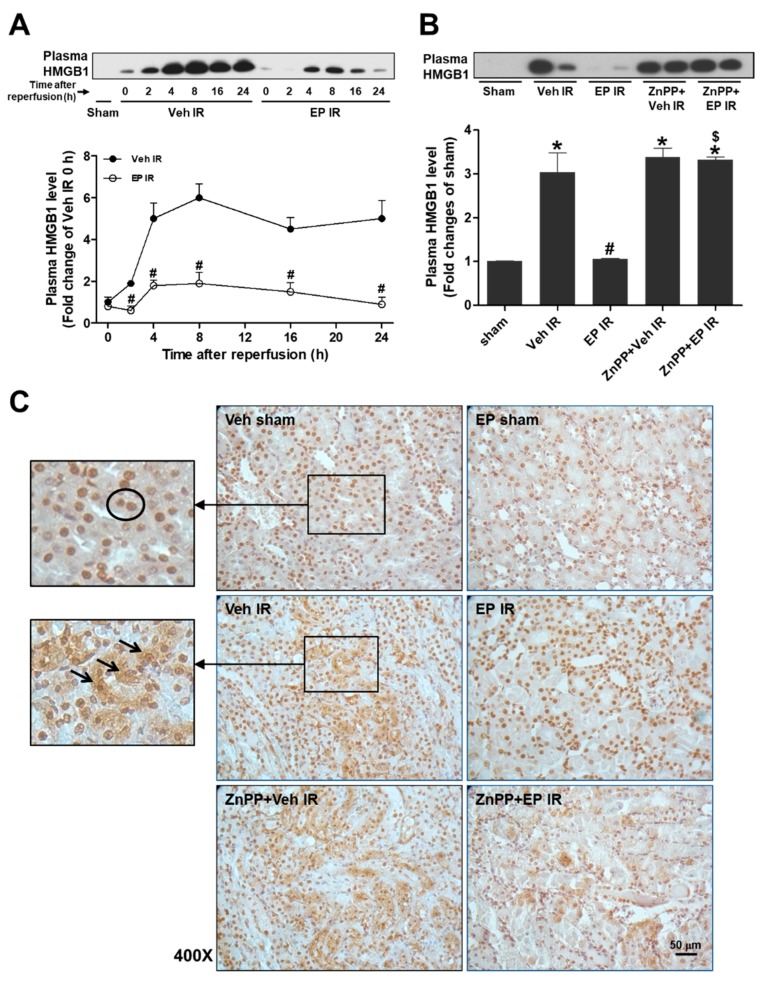
EP inhibits HMGB1 secretion during renal IR. Mice were subjected to either a sham-operation or 25 min of renal ischemia and subsequent reperfusion. EP (40 mg/kg) and ZnPP (10 mg/kg) were given i.p. to mice at 1 h and 2 h prior to ischemia, respectively. (**A**) Plasma HMGB1 levels were determined by western blot analysis from the mice at 0–24 h after reperfusion. (**B**) Plasma HMGB1 levels were determined from the mice at 24 h after reperfusion, being treated with EP or ZnPP as indicated. Representative western bands and the quantification were shown. (**C**) Renal sections were subjected to immunohistochemical staining of HMGB1. Higher magnified images present HMGB1-positive proximal tubular cells; a predominant nuclear HMGB1 expression (circled cells) in Sham and EP IR mice, whereas an increased cytosolic HMGB1 expression (arrows) in Veh IR and ZnPP-treated mice. Data are presented as the mean ± SEM. * *p* < 0.05 vs. Veh sham, ^#^ *p* < 0.05 vs. Veh IR, ^$^ *p* < 0.05 vs. EP IR. Scale bar, 50 µm.

To determine the levels of plasma HMGB1 released during renal IR injury, plasma was collected at 2, 4, 8, 16, and 24 h after reperfusion from the mice treated with vehicle or EP. The HMGB1 levels were rapidly increased within 4 h after reperfusion and maintained the levels up to 24 h after reperfusion. EP-treated mice showed a significant reduction in plasma HMGB1 levels and ZnPP pretreatment abolished the EP effect (Figure 3A,B). To examine the HMGB1 expression and localization altered during renal IR injury, renal sections were subjected to immunohistochemical staining with an HMGB1-specific antibody (Figure 3C). IR mice showed a dramatic increase of cytoplasmic HMGB1, whereas sham mice showed a confined expression of HMGB1 in the nucleus (in Figure 3C insets). EP significantly reduced the cytoplasmic translocation of nuclear HMGB1, but the effect of EP was blocked by ZnPP pretreatment. These results indicate that EP reduced IR-induced HMGB1 secretion through HO-1. 

### 3.4. EP Significantly Increases Tubular HO-1 Expression during Renal IR Injury

**Figure 4:**
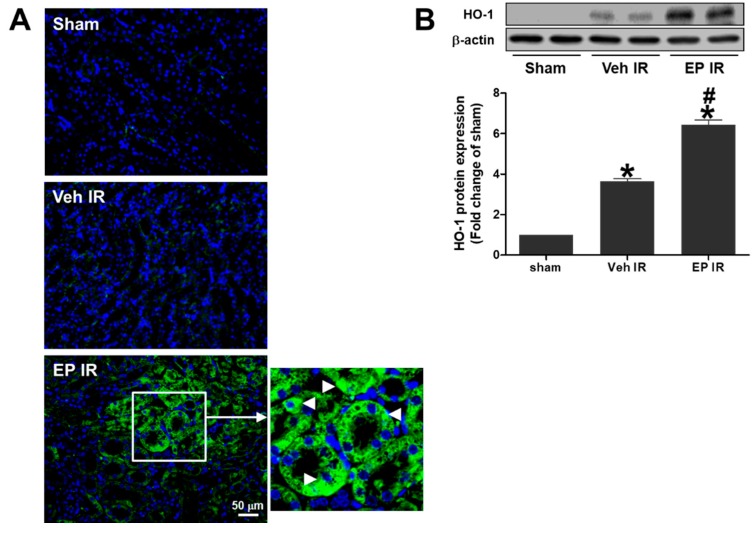
EP induces the expression of HO-1 protein in proximal tubular cells during renal IR. Mice were subjected to either a sham-operation or 25 min of renal ischemia and 24 h of reperfusion. EP (40 mg/kg) or vehicle was given i.p. to mice 1 h prior to ischemia. (**A**) Representative images of HO-1 immunofluorescence staining of renal sections (HO-1, green; nuclei, blue). Arrowheads indicate the HO-1-positive proximal tubular cells. (**B**) HO-1 protein levels were determined by western blotting; representative western blots and the quantification were shown. Data are presented as the mean ± SEM. * *p* < 0.05 vs. Veh sham, ^#^ *p* < 0.05 vs. Veh IR. Scale bar, 50 µm.

To determine whether EP causes HO-1 induction during renal IR injury, HO-1 expression was assessed by immunofluorescence staining and western blot analysis using a HO-1 specific antibody (Figure 4). IR mice showed a significant increase of proximal tubular HO-1 expression compared to sham mice, and EP-treated mice showed a further HO-1 induction (arrows in Figure 4A inset). Renal HO-1 protein expression was increased (by about 3.6 times that of sham) at 24 h after reperfusion, and further enhanced (by about 6.4-fold that of sham) by EP. The results indicate that EP attenuated renal IR injury by inducing tubular HO-1 expression.

### 3.5. EP Increases HO-1 Expression in Proximal Tubular HK-2 Cells in Vitro

**Figure 5:**
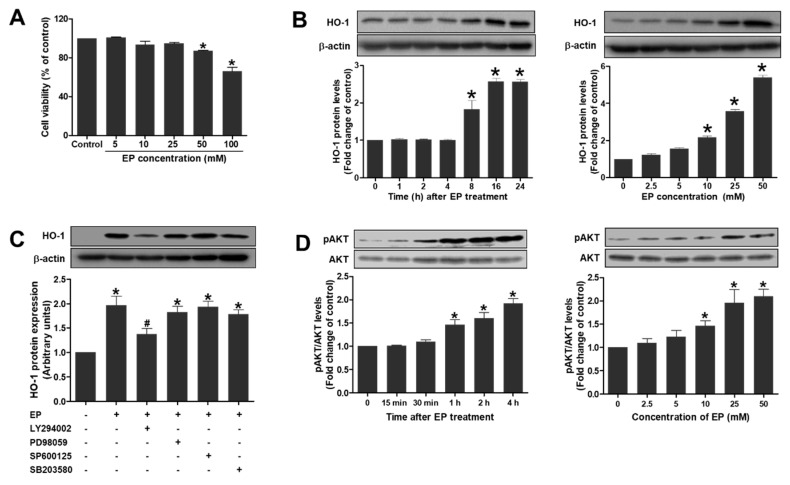
EP induces HO-1 protein expression by activating PI3K/Akt pathway in HK-2 cells. (**A**) Cell viability was determined by MTT assay in the cells treated with EP (0–100 mM) for 24 h. (**B**) Cells were treated with EP (25 mM) for indicated times (0–24 h), or treated with EP at various concentrations (0–50 mM) for 24 h. (**C**) Cells were pretreated with LY294002 (PI3K inhibitor, 10 µM), PD98059 (ERK inhibitor, 50 µM), SP600125 (JNK inhibitor, 40 µM), or SB203580 (p38 inhibitor, 10 µM) for 1 h, and then treated with vehicle or EP (25 mM). HO-1 protein levels were determined by western blot analysis. (**D**) Cells were treated with EP (25 mM) for indicated times (0–4 h), or treated at various concentrations (0–50 mM) for 4 h. p-Akt and Akt levels were determined by western blot analysis. Representative western blots and the quantification were shown. Data are presented as the mean ± SEM. * *p* < 0.05 vs. Veh control, ^#^ *p* < 0.05 vs. EP.

To investigate the molecular mechanism of EP through HO-1 induction during renal IR injury, human proximal tubular HK-2 cells were treated with EP and further assays were performed. First, HK-2 cells were treated with EP at concentrations of 5–100 mM and subjected to MTT assay. EP showed no cellular toxicity at concentrations of up to 25 mM, but caused a significant decrease in cell viability at 50 and 100 mM (Figure 5A). Thus, EP was treated at 25 mM in subsequent in vitro experiments. To determine the effect of EP on HO-1 expression, HK-2 cells were treated EP (25 mM) for various times (1–24h) or treated with various concentrations of EP (2.5–50 mM) for 24 h (Figure 5B). EP significantly induced HO-1 protein levels in a time- and dose-dependent manner. The results are consistent with in vivo study, showing that EP caused HO-1 induction in the proximal tubular cells.

### 3.6. EP Induces HO-1 Protein Expression by Activating PI3K/Akt Pathway in HK-2 Cells

To determine whether mitogen-activated protein kinase (MAPK) signaling is involved in HO-1 induction caused by EP, the specific inhibitors were pretreated as follows; LY294002 (PI3K inhibitor), PD98059 (ERK inhibitor), SP600125 (JNK inhibitor), or SB203580 (p38 inhibitor). EP increased the HO-1 protein levels, which were significantly inhibited by LY294002, but not by PD98059, SP600125 or SB203580 (Figure 5C). In addition, EP increased the phosphorylated Akt (p-Akt) levels significantly at 25 mM concentration for 1–4 h of treatment, or at 10–50 mM for 4 h (Figure 5D). These results indicate that EP increased HO-1 expression by activating PI3K/Akt pathway in HK-2 cells.

### 3.7. EP Induces HO-1 Protein Expression through Nrf2 Activation in HK-2 Cells

**Figure 6:**
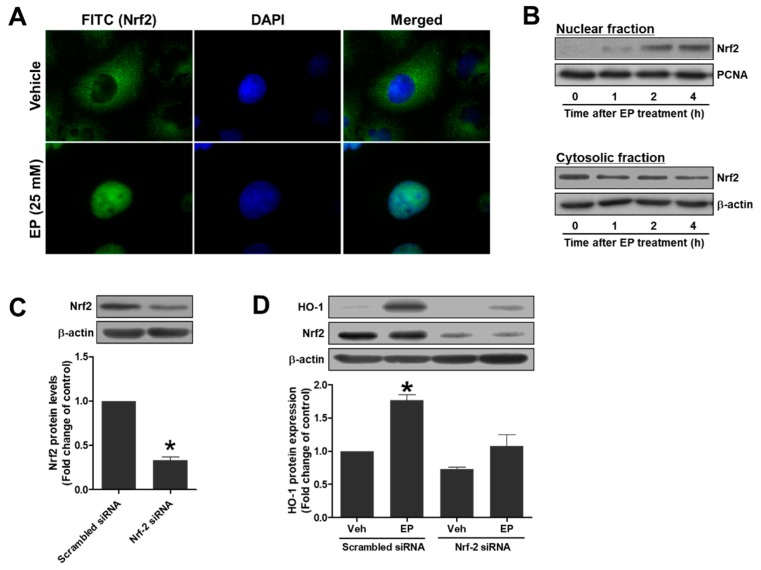
EP induces HO-1 protein expression through Nrf2 activation in HK-2 cells. (**A**) Cells were treated with EP (25 mM) for 4 h and subjected to immunofluorescence staining (Nrf2, green; nuclei, blue). (**B**) Cells were treated with EP (25 mM) for indicated times (0–4 h), and nuclear and cytosolic fractions were separated for western blot analysis. (**C**) Cells were transfected with scrambled or Nrf2-specific siRNA and Nrf2 protein levels were determined by western blot analysis. (**D**) Cells were transfected with scrambled or Nrf2-specific siRNA, and treated with vehicle or EP (25 mM) for 24 h. HO-1 and Nrf2 protein levels were determined by western blot analysis. Representative western blots and the quantification were shown. Data are presented as the mean ± SEM. * *p* <0.05 vs. Scrambled siRNA control.

To determine whether EP induces HO-1 expression through Nrf2 activation, subcellular localization of Nrf2 was assessed in EP- or vehicle-treated HK2 cells. Immunofluorescence staining of Nrf2 showed that EP (25 mM) markedly increased nuclear Nrf2, but reduced cytoplasmic Nrf2 in HK-2 cells (Figure 6A). Nuclear-to-cytoplasmic translocation of Nrf2 was also determined by western blotting after isolating nuclear and cytosolic proteins. EP-treated cells showed a prominent nuclear Nrf2 expression compared with vehicle-treated cells (Figure 6B). To reveal whether Nrf2 is required for HO-1 induction by EP, the cells were transfected with Nrf2-specific siRNA (Figure 6C). HO-1 expression was significantly increased by EP in the cells transfected with scrambled siRNA; however, HO-1 induction by EP was blocked in the cells transfected with Nrf2-specific siRNA. The results indicate that Nrf2 activation was required for HO-1 induction by EP in HK-2 cells. 

### 3.8. EP Attenuates the Active Secretion of HMGB1 Stimulated by TNF-α in HK-2 Cells

**Figure 7:**
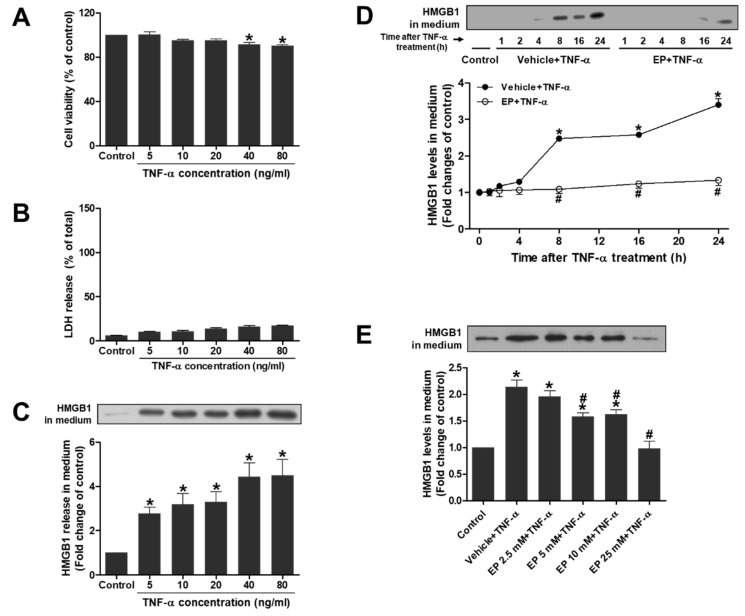
EP inhibits the active secretion of HMGB1 stimulated by TNF-α in HK-2 cells. (**A**,**B**) Cells were treated with TNF-α at indicated concentrations (0–80 ng/mL) for 24 h and subjected to MTT or LDH assay. (**C**) HMGB1 protein levels in media were determined by western blot analysis. (**D**) Cells were pretreated with EP (25 mM) for 1 h, and then treated with TNF-α (10 ng/mL) for indicated times (1–24 h). The HMGB1 protein levels in media were determined by western blot analysis. (**E**) Cells were pretreated with EP at indicated concentrations (0–25 mM) for 1 h, and then treated with TNF-α (10 ng/mL) for 24 h. The HMGB1 protein levels in media were determined by western blot analysis. Representative western blots and the quantification were shown. Data are presented as the mean ± SEM. * *p* < 0.05 vs. Veh control, ^#^ *p* < 0.05 vs. Veh + TNF-α.

To induce active secretion of HMGB1 in HK-2 cells, the cells were treated with TNF-α, a major proinflammatory cytokine involved in early inflammatory response during IR injury. First, the cells were treated with TNF-α at concentrations of 5–80 ng/mL for 24 h and subjected to MTT or LDH assay for cytotoxicity. TNF-α caused no cellular toxicity at concentrations of up to 20 ng/mL (Figure 7A,B). Then, HMGB1 levels in medium were measured at 5–80 ng/mL of TNF-α after 24 h by western blot analysis. Unlike the results from MTT or LDH assay, the HMGB1 levels were significantly increased about 3 folds, starting at 5 ng/mL of TNF-α and the levels were further increased about 5 folds, at 40 and 80 ng/mL of TNF-α after 24 h treatment (Figure 7C). The results indicate that HMGB1 is actively secreted by TNF-α at 20 ng/ml or below before being released from necrotic cells; and thus, TNF-α was treated at 20 ng/mL in subsequent in vitro experiments. To determine the effect of EP on HMGB1 secretion, the cell culture medium was collected at 1–24 h after TNF-α treatment. HMGB1 levels in medium were significantly increased at 8, 16 and 24 h after TNF-α, but these increases were significantly attenuated by EP (Figure 7D). In addition, the HMGB1 secretion was dependent on EP concentrations (2.5–25 mM) where 25 mM of EP reduced the levels of HMGB1 similar to control cells (Figure 7E). These results indicate that EP attenuates the active secretion of HMGB1 induced by TNF-α in HK-2 cells.

### 3.9. PI3K/Akt and HO-1 Induction is Required for EP to Inhibit the HMGB1 Secretion in TNF-α-Treated HK-2 Cells

**Figure 8:**
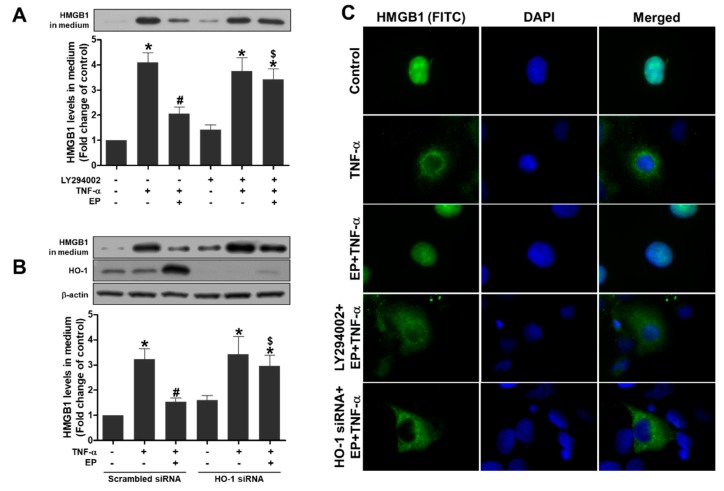
PI3K/Akt and HO-1 induction is required for EP to inhibit the HMGB1 secretion in TNF-α-treated HK-2 cells. (**A**) Cells were pretreated with LY294002 (10 µM) for 30 min, and with EP (25 mM) for 1 h, then were treated with TNF-α (10 ng/mL) for 24 h. (**B**) Cells were transfected with scrambled or HO-1-specific siRNA, and treated with EP (25 mM) for 1 h, then were incubated with TNF-α (10 ng/mL) for 24 h. The HMGB1 or HO-1 protein levels in media were determined by Western blot analysis. Representative western blots and the quantification were shown. Data are presented as the mean ± SEM. * *p* < 0.05 vs. Veh (or Scrambled siRNA) control, # *p* < 0.05 vs. Veh + TNF-α, ^$^ *p* < 0.05 vs. EP + TNF-α. (**C**) Representative images of HMGB1 immunofluorescence staining were shown (HMGB1, green; nuclei, blue).

To investigate the mechanism of EP inhibiting the HMGB1 secretion induced by TNF-α, HK-2 cells were pretreated with LY294002 (PI3K inhibitor) or HO-specific siRNA. As shown in Figure 8A, EP inhibited the HMGB1 secretion induced by TNF-α, but this was blocked by a pharmacological inhibition of PI3K. The inhibition of HMGB1 secretion by EP was also blocked by a genetic depletion of HO-1 using HO-1-specific siRNA (Figure 8B). The translocation of HMGB1 from nucleus to cytoplasm induced by TNF-α was examined by immunofluorescence staining using HMGB1 antibody. EP blocked the HMGB1 translocation, and the effect of EP was abrogated by LY294002 or HO-1-specific siRNA (Figure 8C). These results indicate that the effect of EP on inhibiting the HMGB1 secretion stimulated by TNF-α depended on PI3K/Akt and HO-1 induction in HK-2 cells.

## 4. Discussion

**Figure 9:**
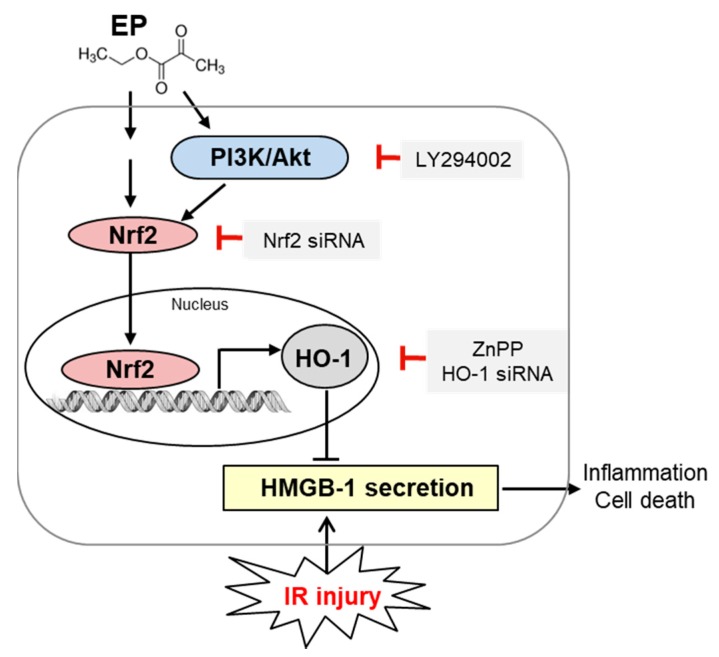
A proposed molecular mechanism of EP that protects the kidney against renal IR injury. EP inhibits the active secretion of HMGB1 from proximal tubular cells during IR injury through HO-1 induction. HO-1 is a cytoprotective enzyme that reduces plasma HMGB-1 followed by tubular inflammation and cell death. EP activates PI3K/Akt and Nrf2 pathway to induce HO-1 expression; LY294002, Nrf2 or HO-1 siRNA, and ZnPP were used to block each signaling molecule/pathway as indicated, to study the mechanism of EP in this study.

The present study showed that EP attenuates renal IR injury by inhibiting the active secretion of HMGB1 through HO-1 induction. EP reduced plasma Cr levels, neutrophil infiltration and inflammatory cytokines and tubular cell death during IR injury. The proximal tubular cells are particularly vulnerable to IR injury, and in these cells severe necrotic and inflammatory responses occur at early IR injury. We showed that EP inhibited the active secretion of HMGB1 from proximal tubular cells in the mice subjected to IR in vivo and the HK-2 cells treated with TNF-α in vitro. EP exerts the renal protection through HO-1 induction because ZnPP pretreatment abolished the effect. In addition, we showed that EP increased HO-1 expression via PI3K/Akt and Nrf2 pathway. Briefly, the important finding of our study is that first, EP attenuates the active secretion of HMGB1; second, HO-1 mediates the effect of EP by inhibiting the HMGB1 secretion; and third, EP induced HO-1 via PI3K/Akt and Nrf2 pathway (Figure 9).

The pathophysiology of renal IR injury includes an initial cellular damage caused by ischemia and a delayed injury from inflammatory responses following reperfusion [10]. The inflammatory initiators or propagators have been actively investigated and recent studies have identified the damage-associated molecular pattern HMGB1, as one of the initiators during IR, propagating inflammatory signaling and exacerbating kidney damage after an ischemic insult [24]. HMGB1 is a nuclear protein that binds to chromatin for structural stabilization and transcriptional regulation [9]. Extracellular HMGB1 functions as a potent inflammatory cytokine through acting on its target receptors such as TLR2, 4, and RAGE. HMGB1 is passively released from necrotic cells or actively secreted from activated immune cells, such as macrophages or dendritic cells; active secretion of HMGB1 is triggered by external stimuli such as endotoxin LPS, TNF-α, or IFN-γ [9]. In systemic inflammation of sepsis, HMGB1 is a late mediator of lethality; however, HMGB1 also acts an early mediator of inflammatory responses following IR. Thus, inhibition of extracellular HMGB1 significantly prevents septic lethality and also protects from IR-induced tissue damage [10,25]. 

In the present study, the plasma HMGB1 levels were rapidly elevated at early reperfusion and nuclear HMGB1 was translocated into cytoplasm in proximal tubular cells, which were inhibited by EP. In hepatic IR injury, HMGB1 levels are elevated in the liver, 1 h after reperfusion and time-dependently increased up to 24 h; in myocardial IR injury, HMGB1 levels are increased in the heart, 30 min after ischemia and significantly high 7 days after IR [26,27]. After renal IR, HMGB1 is translocated from nucleus to cytoplasm and released extracellularly; plasma HMGB1 was significantly elevated from 1 h and maintained up to 24 h after reperfusion [28], which is consistent to our study. The plasma Cr, the degree of renal dysfunction is correlated with HMGB1 increase after IR, supporting that HMGB1 is a critical mediator of inflammation. 

HO-1 is induced by various stimuli and considered as a sensitive indicator of cellular stress. HO-1 upregulation is an adaptive mechanism that protects cells from stress, such as hypoxia, ischemia, or inflammation [29]. Macrophages expressing HO-1 protects renal structure and function from IR injury [30]. Recently, EP is reported to induce HO-1 through p38 MAPK by depleting glutathione in LPS-activated macrophage cells [23]. The present study showed that EP1 increased HO-1 through PI3K/Akt and Nrf2 pathways and inhibited the HMGB1 secretion in proximal tubular cells. The effect of EP was abrogated by ZnPP, suggesting HO-1 plays an essential role in the protective mechanism of EP. HO-1 levels in the kidney were increased during renal IR in vehicle-treated mice, and further elevated by EP. Immunostaining of HO-1 in kidney tissues also showed a strong induction of HO-1 by EP, mostly in proximal tubular cells, most susceptible to renal IR injury. Consistently, HO-1 expression was dramatically increased by EP in HK-2 cells in vitro, where the study of EP-mediated signaling was conducted. 

The present study showed that EP1 increased HO-1 through PI3K/Akt and Nrf2 pathways because the effect of EP was abrogated by a PI3K inhibitor or Nrf2-specific siRNA. NF-E2-related factor 2 (Nrf2) is a transcription factor that regulates antioxidant and cytoprotective genes; Nrf2 is complexed with Keap1, and dissociated upon various stimuli, then translocated to the nucleus to act as a transcriptional activator of antioxidant genes, including HO-1 [31]. Consistently, a depletion of Nrf2 in mice increases susceptibility to cisplatin- or IR-induced renal injury [32].

Pyruvate is a key intermediate in energy metabolism, but the renal cortical pyruvate has been depleted in mice subjected to ischemia or glycerol-induced AKI; pyruvate administration confers a marked protection against AKI by increasing anti-inflammatory IL-10 and HO-1 levels [33,34]. Pyruvate has been reported to have anti-oxidant and anti-inflammatory properties [35,36]. Pyruvate attenuated organ injury or dysfunction in animal models of intestinal, hepatic, or renal IR injury [37,38,39]. Despite these findings, the development of pyruvate as a therapeutic drug has been limited due to poor stability in aqueous solution [21]. Interestingly, EP, a simple ester of pyruvic acid, is more stable than pyruvate, having pharmacological effects; EP decreases sepsis-induced AKI and prevents lethality in mice [22,40]. EP ameliorates IR-induced intestinal mucosal injury and hemorrhagic shock [41,42]. Consistently, the present study showed that EP has anti-inflammatory, anti-necrotic and anti-apoptotic effects against renal IR via HO-1 induction and EP effectively inhibits the HMGB1 secretion. Recent studies reported that EP markedly decreased the HMGB1 release in murine colitis, sepsis or renal IR [22,43,44]. Consistently, our data showed that EP inhibits the HMGB1 secretion induced by renal IR or TNF-α, especially in proximal tubular cells.

## 5. Conclusions

Taken together, EP inhibits the active secretion of HMGB1 from proximal tubular cells following IR injury through HO-1 induction, which is an important protective mechanism; the HO-1 induction by EP depends on PI3K/Akt and Nrf2 pathways (Figure 9). We here propose that EP is a potent therapeutic drug to protect from the proximal tubular injury at early reperfusion during renal IR.
